# Penicillin-resistant gonorrheal endocarditis presenting with large annular abscess and aortic valve destruction

**DOI:** 10.1093/jscr/rjad212

**Published:** 2023-05-29

**Authors:** Abhishek K Kashyap, Hamza Abu-Hussain, Raja GnanaDev, Ahmed Seliem, Reza Salabat

**Affiliations:** Department of Cardiothoracic Surgery, Loma Linda University, Loma Linda, CA, USA; Department of Cardiology, Loma Linda University, Loma Linda, CA, USA; Department of Surgery, Arrowhead Regional Medical Center, Colton, CA, USA; Department of Cardiology, Loma Linda University, Loma Linda, CA, USA; Department of Cardiothoracic Surgery, Loma Linda University, Loma Linda, CA, USA

## Abstract

Disseminated gonorrheal infections with cardiac involvement are rare, with fewer than 100 cases reported. The increasing prevalence of gonococcal infections and increasing antibiotic resistance represent a concerning challenge to public health. Here we report a case of antibiotic-resistant gonococcal endocarditis presenting with cardiogenic shock and discuss principles of diagnosis and treatment.

## INTRODUCTION

Gonorrheal infections caused by *Neisseria gonorrhoeae* are the second most commonly reported sexually transmitted disease in the United States with a total of 677 769 reported cases in 2020. The prevalence of gonorrhea and reported cases have increased by 111% since hitting a nadir in 2009 [1]. This trend has continued even through the ‘shelter-in-place’ mandate implemented during the COVID-19 pandemic, with preliminary data from the CDC showing 696 764 reported cases in 2021 [2]. Gonorrheal disease is most common among young, sexually active adults between 15 and 24 years of age. Disseminated gonorrheal infections are uncommon, representing ~1–2% of all cases. Of these cases, 1–2% will present with gonococcal endocarditis [3]. Mortality remains high for disseminated gonorrheal infections, around 19–20% [4]. Antibiotic resistance is similarly increasing and represents a growing and significant public health challenge. Here we present the case of a previously healthy young woman with gonococcal endocarditis.

## CASE REPORT

A 32-year-old sexually active woman with a history of methamphetamine use presented to the emergency department with symptoms of severe shortness of breath. She reported intermittent shortness of breath for the prior 6 months which had significantly worsened the day of presentation. She also reported a 2–3 day history of watery diarrhea with generalized abdominal pain and sharp, substernal non-radiating chest pain.

Vital signs showed blood pressure of 130/40 mm Hg, heart rate of 134 beats per minute, respiratory rate of 37 breaths per minute, oxygen saturation of 75% on room air with a temperature of 38.5°C. On physical examination, she was in visible respiratory distress and agitated. A 2/6 blowing murmur was appreciated along the left sternal border at the third intercostal space. Pelvic examination showed thick, yellow-white discharge. Laboratory evaluation showed white blood cells 27.5×10 [4]/μl, platelets 482, troponin 0.52 ng/ml, lactate 3.15 mmol/L, B-type natriuretic peptide 10 483 pg/ml, urinalysis with 3+ leukocyte esterase, positive nitrites, 2+ trichomonas, 2+ bacteria, 50–100 WBC/HPF, negative respiratory viral panel including SARS-COV2. Chest CT showed no evidence of pulmonary embolism but demonstrated large bilateral pleural effusions.

She was intubated due to respiratory failure and septic shock and admitted to the intensive care unit on norepinephrine and vasopressin. She was started empirically on cefepime, metronidazole and trimethoprim-sulfamethoxazole due to a severe vancomycin allergy. Four sets of blood cultures were drawn and all subsequently grew *N. gonorrhoeae* which was resistant to penicillins and fluoroquinolones but susceptible to third generation cephalosporins.

**Figure 1:**
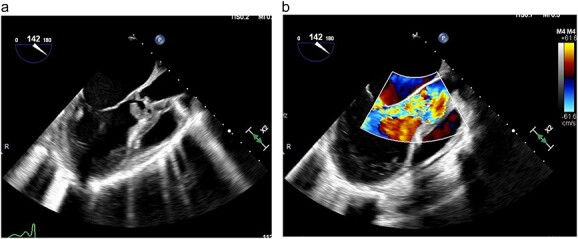
(**a**) TEE demonstrating large vegetation under aortic valve, (**b**) TEE demonstrating severe aortic regurgitation.

A transthoracic echocardiogram was thus performed and demonstrated normal left ventricular function, severe aortic regurgitation, a 2.33 x 1.89 cm vegetation on the aortic valve, moderate mitral regurgitation and severely dilated left atrium (Fig. 1). The cardiothoracic surgery team was consulted and the patient was taken to the operating room emergently for aortic valve replacement due to severely deteriorating hemodynamic status. Intraoperatively, she was found to have complete destruction of the right and left aortic valve leaflets with preservation of the non-coronary leaflet. The aortic annulus was also destroyed and detached from the left ventricle with a large vegetation under the right leaflet. The aortic root was completely debrided and the annulus was reconstructed with a bovine pericardial patch. Subsequently, an aortic root enlargement was performed and a 23 mm bovine pericardial tissue valve was implanted. Intraoperative gram stain showed rare polymorphonuclear leukocytes with no organisms and subsequent intraoperative tissue cultures showed no growth. Her postoperative course was unremarkable as she was rapidly weaned off vasopressors following surgery and extubated on postoperative Day 5. Due to her social circumstances, she remained inpatient and she was treated with a 6-week course of 2 g IV ceftriaxone and 1-week course of metronidazole for concomitant trichomonas infection. Following this she was discharged to home on hospital Day 42. During her hospitalization, a pelvic ultrasound was completed and did not demonstrate the evidence of pelvic inflammatory disease.

## DISCUSSION

*N. gonorrhoeae* was first discovered in 1879 by a German physician, Albert Neisser, studying patients with urethritis and ophthalmia neonatorum [5] and prior to the advent of antibiotics, it was responsible for up to 26% of all bacterial endocarditis [6]. Currently, disseminated gonorrheal infections are uncommon with gonococcal endocarditis representing an extremely rare presentation [4]. Given the high mortality of disseminated gonorrheal infections, it is important to detect gonococcal infections in the early stages and to keep a broad differential for the cause of endocarditis [4].

The most common presenting symptoms of gonococcal infections are fatigue and fever [7]. Other symptoms may include dysuria/urethral discharge, polyarthralgia, septic joints, skin rash, jaundice if there are hepatobiliary complications, stiff neck or altered mentation with meningitis and new onset cardiac murmurs or dyspnea with valvular involvement. Two-thirds of cases present with no genitourinary symptoms at all, making the diagnosis more elusive [7, 8]. An awareness of these symptoms along with physical exam findings may help with earlier diagnosis and increase the index of suspicion for a gonorrheal cause of endocarditis.

Gonococcal endocarditis is known to be aggressive and frequently presents with valvular vegetations, valve destruction and paravalvular abscess [9]. The aortic valve is most commonly involved (50%) followed by mitral (24.2%) and then pulmonary valve (14.3%). Due to progression to congestive heart failure, more than half of patients with gonococcal endocarditis require valvular surgery [7]. Interestingly, intraoperative cultures are frequently negative despite extensive infections [10].

Growing antibiotic resistance is of particular concern given the severity of disseminated disease with cardiac involvement. Prior to 2007, fluoroquinolones were first-line treatment for gonococcal infections. However, the emergence of fluoroquinolone resistant strains was noted in 2007 [11]. CDC surveillance data continue to demonstrate rising resistance to penicillins, ciprofloxacin and azithromycin [1].

Current recommendations for treatment maintain ceftriaxone as first-line therapy, although antibiotic resistance to third generation cephalosporins has been reported and is increasing in prevalence [12]. Elevated minimum inhibitory concentrations (MICs) to cephalosporins have tripled between 2000 and 2010 from 0.1 to 0.3% [11]. Although the drug resistance to cephalosporins is increasing dramatically, it remains a highly effective treatment. Providers should be cautious of treatment failure, given the severity of disseminated disease with cardiac involvement, and should report such failures to the CDC [12].
